# Standards and Guidelines in Telemedicine and Telehealth

**DOI:** 10.3390/healthcare2010074

**Published:** 2014-02-12

**Authors:** Elizabeth A. Krupinski, Jordana Bernard

**Affiliations:** 1 Department of Medical Imaging, University of Arizona, 1609 N Warren Bldg 211, Tucson, AZ 85724, USA; 2 American Telemedicine Association, Washington, DC 20036, USA; E-Mail: JBernard@americantelemed.org

**Keywords:** telemedicine, standards, guidelines, practice, research

## Abstract

The development of guidelines and standards for telemedicine is an important and valuable process to help insure effective and safe delivery of quality healthcare. Some organizations, such as the American Telemedicine Association (ATA), have made the development of standards and guidelines a priority. The practice guidelines developed so far have been well received by the telemedicine community and are being adopted in numerous practices, as well as being used in research to support the practice and growth of telemedicine. Studies that utilize published guidelines not only help bring them into greater public awareness, but they also provide evidence needed to validate existing guidelines and guide the revision of future versions. Telemedicine will continue to grow and be adopted by more healthcare practitioners and patients in a wide variety of forms not just in the traditional clinical environments, and practice guidelines will be a key factor in fostering this growth. Creation of guidelines is important to payers and regulators as well as increasingly they are adopting and integrating them into regulations and policies. This paper will review some of the recent ATA efforts in developing telemedicine practice guidelines, review the role of research in guidelines development, review data regarding their use, and discuss some of areas where guidelines are still needed.

## 1. Introduction

There is a large body of evidence supporting telehealth’s utility and benefits, but the field spans health, as well as technological, economic, and social/organizational communities. Consequently, there are differences in approaches and norms for conducting telehealth [1,2]. We must reconcile these differences if telehealth is to become an integral part of the health care system [3,4,5]. In any field, improving performance and accountability depends on having a shared goal that unites the interests and activities of all stakeholders. One of those shared goals should be the development of sound clinical practice guidelines. Clinical guidelines development has increased in recent years, and the field of telemedicine is no exception. The guidelines development process relies heavily on research, and the good news is that the existing body of telehealth evidence is now robust enough to create evidence-based guidelines and standards. This is a clear indication of the maturity of the field, as well as the quality and quantity of research that has been conducted.

This paper will provide an overview of practice guidelines in telemedicine. It starts with the role of research as a foundation for the evidence base required for guidelines development, and the various types of studies that are generally conducted to provide the evidence upon which developers base their recommendations. This is followed by a review of the literature regarding the quality of research in telemedicine, highlighting a number of key meta-analyses that have been conducted in recent years. What is still needed in terms of research in telemedicine is addressed as well, followed by a discussion of how research is actually used in the guidelines development process. As a leader in the promotion and support of telemedicine, the American Telemedicine Association’s (ATA) guidelines development process is then summarized, with a subsequent discussion of how and why those in the telemedicine community are using guidelines today. The ways in which the ATA promotes the use of guidelines and how they can be freely accessed are discussed. Finally, a summary and some overall conclusions are provided.

## 2. Study Designs and Telemedicine Evaluation

Despite the diversity in telemedicine, there still exists a core set of study types that can address clinical and scientific data while providing the needed evidence for establishing practice guidelines [2]. The appropriate use of each of these methodologies needs to be considered in the context of timeliness, scope, and reliability [2,3,4]. In depth explanations of study designs can be found in any basic research methods or statistics text, and are nicely summarized in [2] as they relate to telemedicine. Readers are encouraged to review such core texts if they are interested in conducting telemedicine research. Briefly, in order of relative importance and impact, the most powerful studies are systematic reviews and meta-analyses. These are especially useful in the public policy arena as they synthesize different relevant studies, giving policy makers a clear, comprehensive overview of the existing research. Following are best evidence, evidence guidelines, and evidence summaries. These types of systematic reviews and summaries typically appear only as a body of evidence evolves, since scientifically sound studies are needed to conduct higher order analyses. These higher order analyses rely on experimental and observational study designs. 

In experimental studies the telehealth intervention is compared to traditional care (control group) of randomly allocated subjects (Randomized Control Studies or RCTs). Observational studies examine subjects and interventions *in situ*, and a control group may or may not be a part of the study design. RCTs and observational studies have advantages and disadvantages, but both are valid in the long run and have been used in many telehealth studies. Cohort, cross-sectional, case, and case series also have a place in telehealth literature, but are less statistically powerful than RCTs. 

These types of study designs serve different purposes. RCTs are generally regarded as the gold standard, but there is some disagreement about whether they are telehealth-appropriate. RCT strengths include randomization, and the fact that they are typically prospective. RCTs are designed by nature to eliminate, or at least minimize, different types of biases. RCTs also allow for meta-analyses (study of studies) to be conducted because of the quantitative nature of the data that is collected. RCTs are, however, expensive and time-consuming, and randomization can be impractical. For telehealth, a double-blind study is nearly impossible, and there are ethical issues related to withholding a potentially beneficial treatment, especially for patients in rural and/or medically underserved areas where treatment access is limited, or even non-existent.

Cohort studies measure the same characteristic in two different groups that typically differ in only one consideration (e.g., telehealth *vs.* traditional care). Eligibility and outcome assessments are usually standardized. However, these studies are observational and rarely randomized (e.g., patients assigned to a group based on in-home Internet access could introduce bias).

Cross-sectional studies are very common in evaluating traditional medical studies, but have been criticized in telehealth as lacking rigor. These studies often use interviews or surveys, and data are collected at a single point in time, which can be a problem for appropriately framing telehealth’s long-term impact. Sometimes, cross-sectional data collection relies on history or recall, which can introduce bias. While these studies are useful for establishing associations rather than causality, the resulting evidence is still very useful.

Case and case series studies are less rigorous, but still yield useful information. Case studies describe a single case (e.g., how teledermatology impacted one individual in a unique manner). They typically describe rare events, early trends, unusual manifestations, or responses to treatment. Case studies are very useful because they are the “people” stories that are used when trying to sway policy makers. 

Case series studies are a little more powerful as they typically report on 10–30 subjects. They include more detailed descriptions than case studies, include a well-described treatment or intervention, have detailed exclusion and inclusion criteria, and can be prospective or retrospective. The main weakness of this approach is the lack of a comparison group. 

In telehealth’s formative years the majority of research efforts focused on feasibility and technology evaluation, and although these studies are sometimes useful for guidelines development, they certainly do not suffice [2,3,4]. In the past decade or so, however, we have seen a significant increase in RCTs [6], cost analyses [7,8,9,10], and clinical outcome-focused studies [6,11,12]. Encouragingly, the telehealth evaluation field now sees a significant number of higher-order studies that either review a sizable body of literature, or actually conduct meta-analyses if sufficient data exist [6,7,8,9,10,11,12]. In fact there are more and more systematic reviews of the literature and meta-analyses being reported in peer-reviewed publications [6,7,8,9,10,11,12].

## 3. How Good Is Telemedicine Research?

In 2010, Ekeland *et al.* carried out a review of reviews using 80 heterogeneous systematic reviews, and found that 21 concluded telemedicine is effective, 18 found evidence is promising but incomplete, and the rest concluded that evidence is limited and inconsistent [6]. They concluded that problematic areas include the nature of the economic analyses conducted, and the quantification of telehealth benefits for patients. 

This analysis of the economic aspect of telemedicine echoes the research concerns raised by Bergmo, who identified 33 economic evaluations that measured both costs (resource use) and outcomes (non-resource consequences) [7]. It found that economic evaluations in telehealth are highly diverse in both study context and methods. Most studies used multiple outcome measures, and analyzed effects using disaggregated cost-consequence frameworks. Although objectives, study design, and choice of comparators were mostly reported, the majority lacked information on perspective and costing methods with insufficient statistical analyses. This in large part is due to the lack of standard methods to conduct economic analyses of telehealth programs, so each analysis accounts and weighs factors differently, and utilizes different cost models. 

To address the economic evaluation issue, Davalos *et al.* reviewed the literature and developed a very useful set of guidelines for telemedicine benefit-cost analyses [8]. Their key research and methods recommendations include: (1) identify and include both accounting and economic program costs using various stakeholder perspectives; (2) identify outcomes readily converted into monetary values that account for stakeholder perspectives, programs objectives, and program maturity; (3) use current and reliable program data on resource utilization and outcomes; (4) utilize RCTs to provide a gold standard for impact; (5) obtain reliable program, context, and specialty monetary conversion factors to estimate the economic benefits; and (6) collect and analyze long-term cost and outcome data for assessing telehealth initiative sustainability and economic value. 

Bergmo also has some recommendations for telehealth economic devaluations, outlining two ways to extend the research findings and conclusions from a study conducted on a sample population to the population at large [9]. The first suggestion is to use trial designs that better reflects normal patient caseload and everyday practice; the second is to use modeling techniques on existing data to estimate expected costs and outcomes of different alternatives. Modeling is an especially attractive option for informing public policy as it provides the means to systematically alter various key parameters of real and simulated data sets to gauge their impact on well-defined economic outcomes using. These predictions can then be validated by comparing them to actual outcomes. Such analyses would involve much more multi-disciplinary research teams than are typically part of a non-academic telehealth program, however, collaborative research efforts between academia and non-academic programs is critical for the field’s advancement.

One of the most recent reviews of research on telehealth cost effectiveness examined 80 studies from 1990–2010. Thirty-eight of the studies involved cost-consequence analyses, with 15 cost-effectiveness analyses (CEA) and seven cost-utility analyses (CUA) [10]. The review revealed that (1) economic tools are increasingly being used for evaluating telemedicine, but we still need better reporting of methodologies and findings; and (2) there is still little conclusive evidence that telehealth interventions are more cost-effective than traditional health care.

Wootton recently conducted a more outcomes oriented review of RCTs for management of five chronic conditions: asthma, chronic obstructive pulmonary disease, diabetes, heart failure, and hypertension [11]. It included RCTs conducted between 1990 and 2011 that had a control group and used one or more telemedicine interventions. The review yielded more than 1,300 publications, and examined the number of subjects, patient types (e.g., disease) intervention (*i.e.*, type of telehealth), intervention time, and outcomes. The overall value of the interventions was rated based on whether primary or secondary outcomes in the intervention group were significantly better, worse, or equivalent compared with the control group. There were 141 RCTs, in which 148 telemedicine interventions were tested with nearly 37,000 patients. Overall, 108 of the trials were favorable towards telemedicine, while 38 showed no statistical difference between telemedicine and traditional care. In 99% of the studies, telemedicine was as good as or better than traditional approaches to care. 

Ekeland and colleagues conducted a review of systematic reviews to summarize methodologies, discuss knowledge gaps, and make recommendations on methodological approaches for further research [12]. They assessed nearly 1,600 studies, and found 50 that described methods used. From this analysis, they made four recommendations for the development of a stronger evidence base for telehealth. To improve the quality of telehealth research, we need: (1) large, rigorous design-control studies that assess telehealth’s impact (for a possible solution, see Tuerk *et al.* [13]); (2) population, intervention, and outcome measure standardization to reduce heterogeneity and to facilitate meta-analyses; (3) combinations of quantitative and qualitative methods; and (4) more naturalistic methods and settings.

Jackson and McLean evaluated studies for metric reporting (*i.e.*, clinical outcomes, satisfaction, patient quality, and cost), and found that while clinical outcomes and patient satisfaction are reported frequently, other performance metrics such as cost are still rather rarely reported [14]. They note that cost assessments and satisfaction studies are critical for assessing effectiveness of interventions and future success. Neglecting these assessment criteria can create problems when stakeholders consider the fate of telehealth programs, as the information can help identify barriers to adoption, facilitators, and possible methods to remove implementation barriers. More importantly, to fully understand the value proposition of telehealth, it is essential that study designs evaluate quality in *relation* to cost.

Overall, these recent reviews of telehealth research indicate that, although work is still needed in terms of standardizing research methods and agreeing on relevant and useful outcomes metrics, there are more and more quality studies being conducted with the preponderance of evidence supporting the benefits in relation to the key parameters of telehealth cost, outcomes, and satisfaction benefits. In the long-run, what we really need to further support and grow telemedicine use and acceptance is more studies focused on clinical outcomes and quality of life with high validity and reliability so they can be generalized from the relatively small sample cohorts to larger patient populations.

The real strengths of the papers summarized here is that they all use solid techniques in their reviews and appropriate statistical methods for the meta-analyses. These types of studies are often quite difficult to carry out, especially when the literature base is still in the maturing phase as is a good deal of the telemedicine literature. These reviews were all very careful in their selection of key search terms, the databases that were searched, the inclusion and exclusion criteria, and the analysis techniques employed (e.g., forest and funnel plots to assess bias). Hopefully we will continue to see more of these high quality systematic reviews and meta-analyses as more research about telemedicine is published in peer-reviewed journals.

## 4. What Else Is Needed?

Research to date clearly demonstrates that technology-enabled health care is not only feasible but in some cases can be equal to or better than in-person care [15]. Nearly every clinical specialty has been evaluated, and found to benefit from telehealth to some degree, whether it be cost savings, time to treatment as a function of better access to services, or clinical outcomes. One might even argue that, to some extent, telehealth has been held to a much higher standard than traditional medicine and has undergone *more* rigorous evaluations. 

So what makes telehealth so different? In some respects, it is the nature of the medium and the rapidity with which the technology keeps changing. As the technology changes, it is incumbent on the telehealth community to verify the reliability and validity of these technologies before use in routine care, and to establish standards and practice guidelines for their use. However, this takes time, effort, and usually funds, and it is often argued that rigorous evaluation studies are done just as the technology becomes obsolete. One way to perhaps deal with this is modeling, as suggested by Bergmo, at least for cost-benefit analyses [9]. For example, a 2011 study, published in Neurology, modeled the costs associated with telestroke *versus* those that would accrue without telestroke, concluding that “telestroke appears cost-effective when compared with usual care” [16]. A distinct advantage to modeling is that a study can be completed in a relatively short amount of time relative to other evaluation methodologies. This becomes particularly important in standards and guidelines development where timeliness of evidenced-based data is critical. 

One sector that is rich with opportunities for telehealth evaluation studies to be used in guidelines development is the United States Department of Veterans Administration (VA), recognized as a national telehealth leader. The VA has utilized a wide range of modalities of telehealth for a number of years [2]. As a vertically integrated, closed system that is client-centered, forward looking, and results oriented, the VA serves as valuable source for assessing scalable and generalizable best practices. As an example, Jia and colleagues conducted a four-year longitudinal study of the long-term effects of home telehealth on preventable hospitalization use [17]. This study demonstrated that in the VA patient-centered Care Coordination Home Telehealth (CCHT) program, CCHT enrollees were less likely to be admitted for a preventable hospitalization than their non-enrollee counterparts. These findings are some of the first that have systematically examined the extent to which home telehealth programs have a long-term effect on preventable hospitalization rates.

Clear and standard evaluation criteria that would lead to readily accessible and reliable data would benefit the development of practice guidelines. This type of standardization would be especially useful for technology evaluations. One such effort was proposed by Ho *et al.* for assessing mobile teledermatology applications [18]. They developed a set of 13 evaluation criteria that encompassed three key domains: (1) technical specifications; (2) user experience and workflow; and (3) integration and scalability, and validated the framework by comparing two mobile teledermatology applications. The framework and criteria could readily be applied to a variety of telemedicine technology evaluations. Similar evaluation frameworks have been proposed by other groups [4,19,20].

A standard taxonomy would also be useful [21,22,23]. For example, Bashshur *et al.* note that a taxonomy serves as an information management strategy to improve knowledge sharing, facilitate research and policy initiatives, and provide guidance for the orderly development of telehealth [23]. They propose a taxonomy that provides conceptual context for the multitude of concepts in the field such as telehealth, e-health, and m-health. The taxonomy will help provide definitive information about the impact and effects of telehealth with respect to cost, quality, and access. 

## 5. Using Research to Support Standards & Guidelines Development

The American Telemedicine Association has developed a number of practice guidelines, and continues to produce more [24,25]. Important highlights include “Core Standards for Telemedicine Operations”, “Expert Consensus Recommendation for Videoconferencing-Based Telepresenting”, and dedicated specialty guidelines for teledermatology, telepathology, tele-home health, tele-mental health, tele-rehabilitation and tele-ophthalmology. Other professional societies have also developed guidelines for telemedicine, including the American College of Radiology [26,27], the American Academy of Dermatology [28], and the American Medical Association [29]. There are also many international guidelines, such as the European Code of Practice for Telehealth [30], all of which are based on research efforts that validated the technologies being used, assessed practice protocols, and examined relative costs and benefits.

## 6. The ATA Guidelines Development Process

The ATA is not the only body developing standards and guidelines for telemedicine, but it is perhaps the organization with the broadest focus, covering a variety of clinical specialties. For example, radiology has a number of technical and practice guidelines in place for digital image acquisition, storage, transfer and display via Picture Archiving and Communications Systems (PACS) and teleradiology [31,32,33,34,35]; and the Society of American Gastrointestinal and Endoscopic Surgeons has guidelines for the Surgical Practice of Telemedicine [36]. The first set of guidelines from the ATA actually pre-dated the formation of the official ATA Standards and Guidelines Committee, and was created for Telepathology in 1999 [37].

The first truly formal set of ATA practice guidelines were created in 2004 when the ATA Ocular Telehealth Special Interest Group published guidelines specifically addressing diabetic retinopathy. The guidelines covered clinical and administrative issues and provided guidelines for designing and implementing a diabetic retinopathy ocular telehealth clinical care program [38]. This effort created the framework for future efforts in terms of addressing technical, administrative and clinical aspects associated with a particular clinical specialty using telemedicine as a means to deliver patient care. 

The success of the teleocular guidelines led to the creation of a standing guidelines committee and process for developing practice guidelines in the ATA. The first practice guidelines developed by the Standards and Guidelines Committee were the Practice Guidelines for Teledermatology [39]. These were the first to recognize that recommendations need different levels of importance and that guidelines are required whenever *feasible and practical* as determined by the referring clinician practicing under local conditions. Thus, there are some guidelines that “shall” be implemented in order to meet a basic minimum level of operation in order to provide quality telehealth care; some that “should” be adopted for optimal practice if possible; and some that are optional or permissible actions that “may” be adopted to optimize the teleconsult process. 

All of the ATA guidelines incorporate a preamble that broadly states the intent of the document and the manner in which it should be used. Three aspects are worth mentioning. The first is that guideline compliance does not guarantee accurate diagnosis or successful outcomes, recognizing that the practice of medicine is both a science and an art and that the immediate, local circumstances need to be considered in order to best help the patient. Thus, the goal of the guidelines is to assist the clinical practitioner in pursuing a sound course of action to provide effective and safe medical care founded on current information, available resources, and patient needs. The second follows from the first, and notes that the primary care practitioner is responsible for all decisions regarding the appropriateness of a specific procedure or course of action. They must consider all presenting circumstances, and if they choose to use an approach that differs from the guideline it does not imply that the approach varied from the standard of care. Reasonable judgment based on local circumstances and the assessment of what is feasible and practical should be used at all times. Finally, the preamble notes that the guidelines are not designed nor meant to be unyielding requirements of practice and are not meant to serve as or be used to establish a legal standard of care.

**Figure 1:**
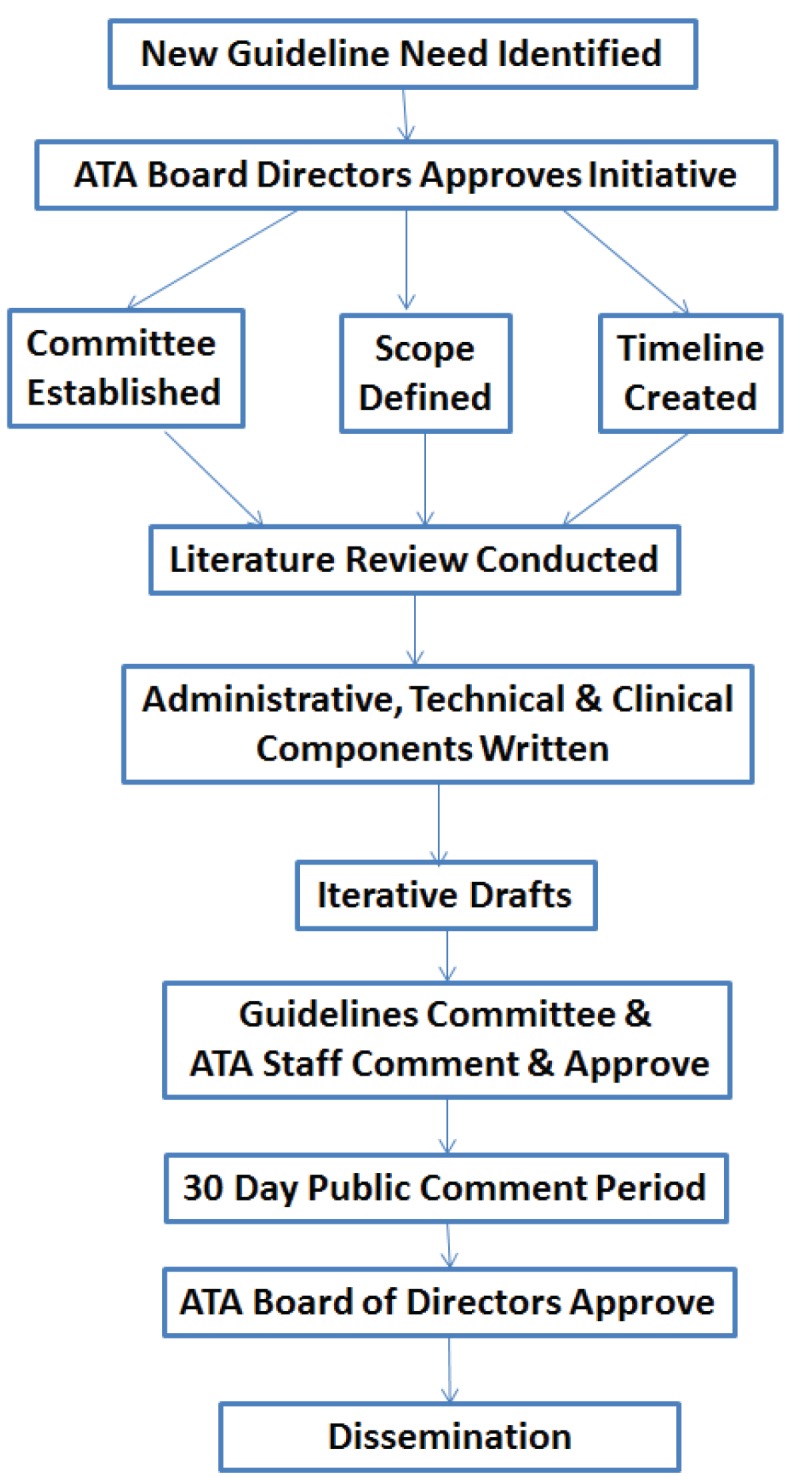
Schematic of the ATA Guidelines development process.

To facilitate the guidelines development process, the Committee has standard operating procedures (Figure 1). The ATA President in consultation with the Executive Committee, appoints a Chair, Vice-Chair, and committee of interested members who serve on a voluntary basis. An ATA senior staff member also serves on the Committee. The Committee meets by phone once a month and at the Annual ATA Meeting. 

The need for a new or revised guideline is either identified by the Committee or by someone in field. The first step is a formal request to the Committee for permission to start the process. If approved, it must then be approved by the Board of Directors. The request includes identification of the guidelines topic/application, who will lead the effort, the names of people who will participate on the working group, and the purpose/rationale, objectives, and area(s) or general scope. The Board of Directors considers the following three principles in rendering a decision: (1) carefully selected priority areas based on adequate documentation and experience in the field; (2) the existence of a gold standard; and (3) a ready constituency willing to actively participate in work groups. Working Groups typically, but not always, emerge from the ATA Special Interest Groups (SIGs). The scope is determined by consensus among the members of the SIG in consultation with the ATA Staff and the Committee. 

After approval, members and chairs of each Working Group are further vetted by the Chair of Standards Committee with the approval by the full Committee. The Working Groups should include all the key stakeholder groups and achieve a balanced representation from clinical, industry, government and other potentially affected parties, both from within and outside the ATA membership. All members sign a disclosure form prior to commencing work on the project.

After the Working Group is formally approved, a timeline is established for the development process (most take 18 months to complete). The process is flexible, but in general begins with an initial meeting (virtual or in-person) in which the Document Scope and Definition are established, identifying any existing clinical benchmark references or gold standards. The definitions and scope serve as the basis for the search terms to be used in the literature review used to establish the scientific basis for the recommendations. This meeting is also often used to delegate tasks and assign roles (e.g., writer, reviewer) for the rest of the process. The writing responsibilities are often divided into three sections—administrative, technical and clinical.

The literature review uses the American Psychiatric Association’s Practice Guideline development Process which codes studies on an A to G scale where A is a randomized, double-blind clinical trial and G is Other (opinion-like essays, case reports) [40]. These study codes are used to formulate the language used in the guidelines, where the key word “shall” indicates a mandatory requirement, statement, or action; while “should” indicates a statement or action that is very highly recommended; and “may” indicates a statement or action that is recommended but optional based on specific scenarios and applications.

Once the literature review is conducted, the Working Group starts to draft the guidelines document, incorporating the literature as appropriate to support the recommendations. The draft undergoes a number of revisions after the Working Group completes its initial draft, including reviews by the Standards and Guidelines Committee and ATA staff. Once a final draft is generated, it is posted on the ATA website for 30 days for public comment, after which it goes to the Board of Directors for approval. Endorsement by external societies is sought and is highly recommended to facilitate adoption by society members. The final documents are posted on the ATA website for free downloading and are published in a peer-reviewed journal. 

**Figure 2:**
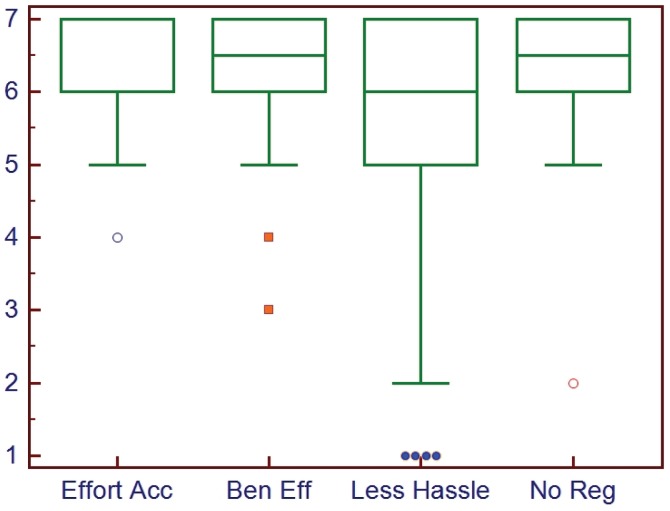
Box plots of the ratings (high score = positive) guidelines developers gave when asked about how well the “think tank” session process went. Effort Acc = effort was acceptable; Ben Eff = they benefitted from the effort; Less Hassle = the process was less of a hassle than expected; No Reg = no regrets.

**Figure 3:**
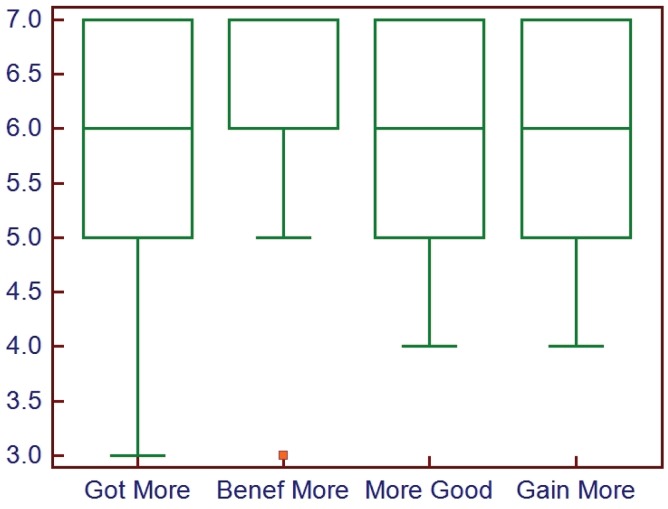
Box plots of the ratings (high score = positive) guidelines developers gave when asked about goals attainment through the “think tank” session. Got more = got more from the session than expected; Benef More = benefitted more from the session than expected; More Good = there was more good derived than expected; Gain More = gained more from the experience than expected.

Recently a new step in the process has been implemented to speed up the generation of the first draft. The key benefit of adding this step is that the ATA has been able to significantly decrease the time it takes to develop the initial document draft, increasing the speed of the overall process and increasing the number of documents developed. Provided is a brief overview of the process and results of an assessment in case others are interested in adopting a similar strategy. After the general scope, definitions and broad outline have been developed by the core members, the Working Group meets in person for a facilitated “Think Tank” session that lasts one to two days depending on the needs of the group. Ideally the group is either very familiar with the existing literature (as they are all experts in the field) or the literature search has been completed for the most part and the literature rating scores available. The session begins with an introductory period to establish the “rules” and allow everyone to get to know each other. Using dedicated “brainstorming” and consensus-building software that allows everyone to anonymously input suggestions to the broad outline, the outline is expanded and key topics inserted. This is followed by a group review to clarify and organize what was added and to identify critical areas that need to be addressed while the entire group is present. After this step the group breaks into pairs and they are assigned specific sections or topics to further develop. The process is iterative and by the end of the session a very solid draft document is generated that can then be completed virtually after everyone returns home. The process has been quite effective and feedback has been positive (Figure 2 and Figure 3).

## 7. Are Guidelines Used and for What Purpose?

Each working group must create a dissemination plan to make the guidelines accessible to as many users as possible and assist practitioners in pursuing a sound course of action to provide effective and safe medical care founded on current information, available resources, and patient needs. Increasingly payers and regulators are utilizing published guidelines to support their policy decisions, thus, extensive outreach to disseminate finished products to these organizations is being done. Posting the guidelines on the ATA website is the key mode of dissemination. The question is whether they are actually used. To asses this, the ATA evaluated a two-year period of guideline downloads. Before being given access (all guidelines are free) to a guideline, requesters fill out a short survey with questions on how they plan to use it, what is the most important information they are seeking, whether their telemedicine program is active, what technologies are used, healthcare sector, healthcare position, and basic demographics (all optional).

In 2012, the ATA recorded over 500 standards document downloads per month. The most common document was the Telemental Health Practice Guideline, accessed about 100 times per month. The survey revealed that most requesters were physicians or PhDs not currently using telemedicine but considering future use. Respondents were mostly from academic health institutions, non-academic medical centers and independent practice, however, government agencies downloaded the documents as well. With respect to how the guidelines were going to be used, most respondents said they would be used for clinical, administrative and research guidance. Other common guidelines downloaded were the telepresenting, diabetic retinopathy, teledermatology, telerehabilitation, and core standards. 

In order to assess awareness, utilization, and impact of the completed ATA standards and guidelines initiatives on the telemedicine industry another survey was conducted [41]. This also served to guide future efforts, as periodic review of clinical guidelines is critical and often incorporates feedback from users in addition to the expert opinions of the developers [42,43,44,45]. The survey had 26 questions, with the majority being yes/no or multiple-choice (could select multiple responses). Contact information was optional. The ATA database was used to send an email blast to 13,177 members and non-members in November, 2011.

**Figure 4:**
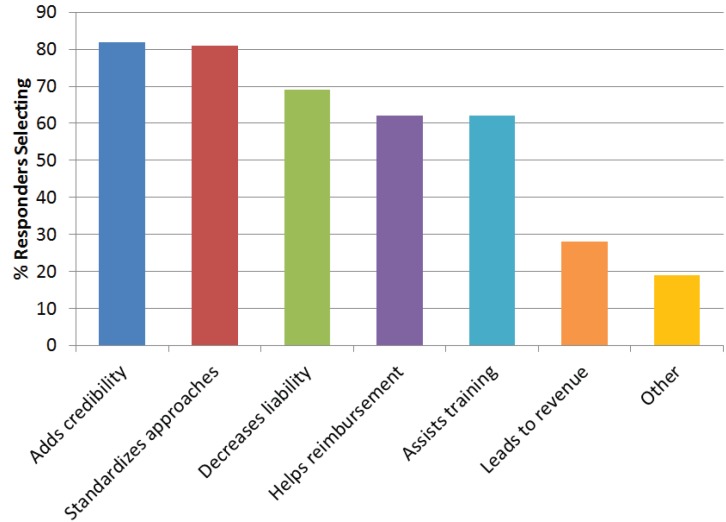
Survey responses to why telemedicine should have guidelines. Responders could provide more than one response.

Seventy-three percent of the respondents were ATA members with only 4% from outside the United States. Most were primarily healthcare providers (31%) and administrators (24%), and were in telemedicine programs that had been active for two to five years or over 10 years. For the majority of questions, responders could select multiple responses. Most (97%) believe that telemedicine/telehealth should have standards and guidelines, primarily because they add credibility, standardize approaches, and decrease liability (Figure 4). Only 4% did not believe there should be standards and guidelines for telehealth, primarily because they do not want outside groups defining quality (59%), feel that standards increase liability (41%), feel they burdensome (41%), too complex to create (29%), not needed (24%), and other (53%) (e.g., telemedicine is no different than traditional practice and guidelines already exist; standards would be a barrier to innovation and growth). Most said the ATA (79%) and other Professional Societies/Associations (75%) should be responsible for development.

**Figure 5:**
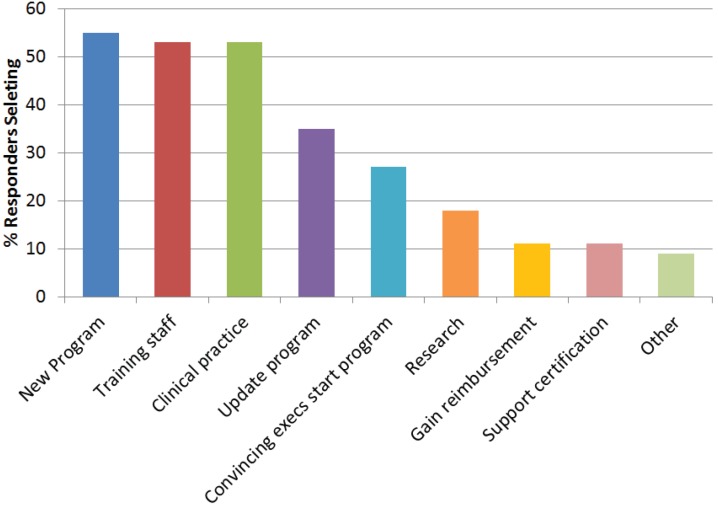
Survey responses regarding top uses of guidelines. Responders could select more than one response.

**Figure 6:**
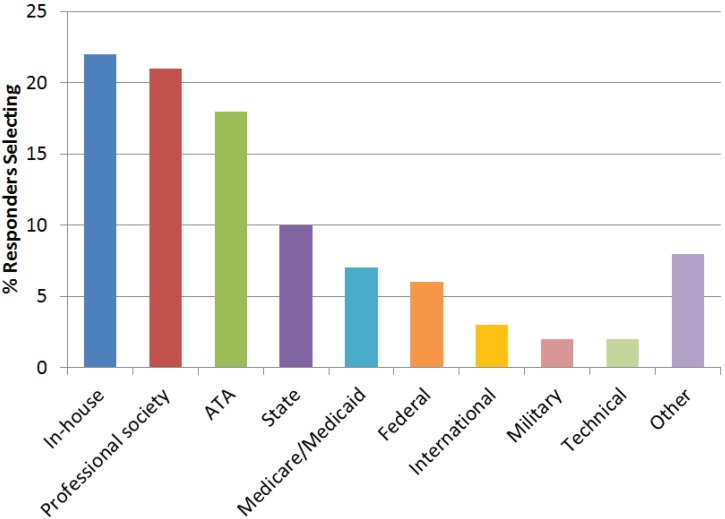
Survey responses as to whose guidelines are typically used. Responders could select more than one response.

The top three uses of guidelines (Figure 5) include setting up a new program (55%), training staff (53%), and clinical practice (53%). Sixty-three percent said they were aware of telehealth guidelines, including the ATA’s (75%), professional associations’ (42%), individual organizations’ (31%), federal agencies’ (27%), state agencies’ (21%), payers’/reimbursers’ (15%), and others (9%) such as the military, NATO, and international bodies. When asked which guidelines are used in their organization for telehealth, most (22%) use in-house (e.g., hospital, company) developed guidelines, those from professional associations/societies (20%), and those from ATA (18%) (Figure 6).

Eighty-six percent said they were aware that there is an ATA Standards and Guidelines Committee, and that they publish standards (86%). Of these, 64% said they use ATA’s guidelines with the Core Standards being used most frequently (58%). Guidelines are used to guide clinical activities (71%), administrative activities (61%), technical aspects (49%), training activities (49%) research (19%), and other purposes (11%). When asked what impact guidelines have on the way they provide healthcare, responses included improved provider acceptance and understanding of telehealth (80%), improved patient outcomes and quality of care (63%), improved access (45%), provides information to justify reimbursement for payers (37%), and reduces costs (26%). 

Gaps where guidelines are still needed include specialty applications such as stroke, home health, teleprescribing/pharmacy, mobile health, primary care, pediatrics, emergency medicine, acute & urgent care, teleICU, reimbursement, technical, and interstate practice. This is surprising since guidelines do exist for some of these. The ATA has guidelines for the general practice of telemedicine, telemental health, teledermatology, home telehealth, telerehabilitation, and teleocular; the American College of Radiology has teleradiology guidelines, and the Centers for Medicare and Medicaid Services has guidelines for e-prescribing. Users may indeed be unaware of existing guidelines, or they may be aware of them but feel they do not address the topic adequately. Telerehabilitation for example covers a number of applications and the existing guideline provides more of a global set of guidelines not specific for example to speech pathology.

## 8. Promoting Effective Use of Guidelines

The ATA clinical practice guidelines are regularly accessed and used, although nearly an equal number of respondents use guidelines developed in-house. One likely reason is that published guidelines for every clinical scenario and application simply do not exist so by necessity are developed in-house. It may also be that even though practitioners feel there is a need for telemedicine practice guidelines, it has been found that clinical practice guidelines generally have a limited impact in changing clinicians’ behaviors. Practitioners may feel uneasy with guidelines developed outside their organization as they may only be applicable in a general sense or difficult to integrate into the local environment and workflow [46,47,48,49]. Key to effective adoption of clinical practice guidelines is to adapt them to the local context and situation [50]. It appears that practitioners of telemedicine are doing just this by developing or adapting existing standards to in-house practice applications.

It is difficult to determine whether or not guidelines are actually utilized beyond performing surveys such as the one described. For example, one recent literature review evaluated strategies to implement clinical guidelines for chronic disease management in primary care in European Union Member States [51]. Five databases were searched for studies assessing management of chronic diseases in adults in primary care, and 21 studies were found that fit the selection criteria. The results indicated that guideline implementation strategies were fully effective in only four (19%), partially in eight (38%), and not effective in nine (43%) reports. Only one of the studies had data on barriers to implementing guidelines, noting primarily a lack of awareness and agreement about clinical guidelines. 

Clinical guideline use is also influenced by the nature and characteristics of the guidelines. A recent study evaluated use of 47 guidelines and found that the recommendations were followed in about 61% of decisions [52]. Recommendations that were controversial were followed only 35% of the time, as were those that were vague and non-specific (36%). When recommendations required changes in existing routines, they were followed only 44% of the time. Evidence-based recommendations were used more (71%) than those not based on research.

Although perhaps not directly related to the adoption of guidelines *per se*, there are always the traditional barriers to adopting telemedicine in general that obviously prevent adoption of guidelines, including the cost of implementation, poor reimbursement for many clinical encounters (although more states are passing parity legislation that requires telemedicine encounters to be reimbursed at the same rate as face-to-face encounters), and lack of regulatory incentive in many venues. 

## 9. Accessing ATA Guidelines

ATA guidelines are free to download and can be found on-line at the ATA website. They include the Quick Guide to Store-Forward and Live-Interactive Teledermatology for Referring Providers, Expert Consensus Recommendations for Videoconferencing-Based Telepresenting, Telehealth practice Recommendations for Diabetic Retinopathy, A Blueprint for Telerehabilitation Guidelines, Practice Guidelines for Videoconferencing-Based Telemental Health, Evidence-Based Practice for Telemental Health, Core Standards for Telemedicine Operations, Practice Guidelines for Teledermatology, Home Telehealth Clinical Guidelines, and Clinical Guidelines for Telepathology. The guidelines are periodically updated and the Committee develops an average of two new guidelines each year. Some of those in the pipeline over the next year or two include tele-burn, teleICU, tele-primary and urgent care, remote data monitoring, an update of telepathology, and tele-audiology for infants screening. Recently the ATA has received support from NIST (National Institute of Standards and Technology) to aid in the development of new guidelines, attesting to the importance of this development from the federal/national perspective. 

There are a number of other professional societies that develop guidelines for telemedicine. Their websites typically provide free access to their guidelines and the majority is also published in relevant peer-reviewed journals. For example, the American College of Radiology has a number of guidelines that relate to the practice of teleradiology some of which were developed in association with the American Association of Physicists in Medicine (AAPM) and the Society for Imaging Informatics in Medicine (SIIM) [26,27,31,32,33,34,35]. As radiology was one of the first clinical specialties to successfully go “tele”, it is not surprising that the radiology community developed and adopted these standards. It is also not surprising that these guidelines specify many more technical requirements that many of the ATA guidelines, given the technology oriented specialty that radiology is. 

It is also important to recognize that the U.S. is not the only country developing telemedicine guidelines. For example, the European Union has a guidelines document available [29] that to some extent provides the same general recommendations that the ATA Core Standards provides, without dealing with individual sub-specialty applications. 

## 10. Conclusions

Practice guidelines for telemedicine are important and valuable for helping insure effective and safe delivery of quality healthcare. Existing practice guidelines have been well received by the telemedicine community and are being adopted in numerous practices. In fact, there are some state medical boards that have specifically incorporated ATA practice guidelines into their rules or proposed rules, including New York, North Carolina, Oklahoma, and Pennsylvania. Basically they state that physicians must practice telemedicine in compliance with standards endorsed by the ATA. 

References to practice guidelines are also appearing in the research literature. For example, Rudnisky *et al.* conducted a validation of a teleophthalmology system incorporating the Diabetic Retinopathy Practice Guidelines for acquisition and display of the images, and for diagnostic grading [53]. Radiology studies nearly always use published standards for the electronic display of images [54,55,56]. Using published guidelines in research not only brings them into greater public awareness, they also provide evidence needed to validate them and guide revisions. 

As already noted, many users of telemedicine guidelines indicate that they have adopted various guidelines because they reduce liability. Guidelines are inherently developed to improve patient safety, but they are not legal documents. In fact, many of them explicitly state that they are not legal documents and should not be used as such, but in reality one could readily foresee legal cases being advanced where guidelines are referred to with the implication that they are binding and legal standards. To date there have been no known cases where telemedicine guidelines have been incorporated into a legal case, but one certainly will arise—hopefully to provide evidence regarding the safety and efficacy of telemedicine rather than the opposite. It is much better to have guidelines than non-existent or poorly defined standards. Without standards, judges and juries are left to make important decisions with their only guidance coming from attorneys who may be biased or simply uneducated about the real possibilities and benefits of telemedicine.

Hopefully this paper has provided readers with the knowledge and background required to (a) understand the way in which practice guidelines for telemedicine are developed; and (b) appreciate the value of having practice guidelines available to promote telemedicine and ensure safe and effective practice. As noted earlier, the goal of having guidelines is not to force everyone to use telemedicine in exactly the same way in every situation with every patient. Guidelines are developed to educate users about the benefits and limitations of telemedicine and to provide them with a set of recommendations about what, based on published evidence, are the most effective, efficient and safe ways to provide patient care incorporating telemedicine technologies and methods. 

It would be interesting to develop a method that would better assess the utility and use of the ATA guidelines. The surveys conducted to date have essentially been sent only to members of the ATA and those that have attended ATA meetings. To some extent these responders are already telemedicine converts and are more aware of the availability of the guidelines and in what ways they are used and are useful. As telemedicine grows, the question is whether the guidelines will be adopted by users less familiar with the ATA or will there continue to be a proliferation of protocols and guidelines developed in-house and/or by other professional organizations? It is impossible to predict. In reality, it is less important that users adopt the guidelines developed specifically by the ATA than to follow standard procedures in general that incorporate the minimum standards required to provide safe and effective care to patients. As with all of medicine, telemedicine is not only science but also an art, and as such the best treatment of an individual patient in his/her unique environment relies upon the judgment and professional experience and expertise of the clinician. The guidelines are there to facilitate that process.

What does the future hold? For the near future, we will continue to need guidelines developed for individual sub-specialties. To some extent this is needed to help convince payers and legislators that telemedicine is just another way to provide patient care and thus should be reimbursed just like any other medical encounter without excessive regulations. Guidelines also help define current limitations to the practice of telemedicine, although in the future it is likely that many of these limitations will fade as even newer technologies emerge (e.g., tools that make remote real-time palpation with haptic feedback a reality so clinicians can “touch” patients). 

Telemedicine guidelines are also going to see more and more incorporation of recommendations for patients. Mobile technologies, apps and other digital tools are being increasingly used by patients in a wide variety of healthcare scenarios and they expect their healthcare providers to accommodate these devices, tools and data into their diagnostic and treatment protocols. There are however limits to what is feasible and practical, and guidelines can help define those limits. For example, should patients expect healthcare providers to diagnose every skin condition captured with a SmartPhone camera? Can patients just Skype their psychiatrist from home whenever they want? These are just a couple of the types of questions starting to arise in the healthcare arena. Whether or not we need guidelines will certainly be addressed soon, but it is clear that at the very least patients and providers need to be educated about what to expect when various technologies are used—what is feasible and appropriate and what the limitations are. The most recent ATA telemental health guideline actually does incorporate some guidance for patients, and the remote data management guideline being developed will as well [25].

In conclusion, telemedicine is clearly here to stay and will continue to grow as an important and viable method for improving access to healthcare throughout the world. Ideally there should not be separate guidelines for providing healthcare services in the traditional manner *versus* telemedicine, but for the present time guidelines do serve a variety of very useful functions and, thus, will continue to have a place in telemedicine.
